# Interaction between mitochondrial NADH dehydrogenase subunit-2 5178 C > A and clinical risk factors on the susceptibility of essential hypertension in Chinese population

**DOI:** 10.1186/s12881-019-0838-3

**Published:** 2019-07-05

**Authors:** Xi Chen, Xiang-Yu He, Chao Zhu, Yusong Zhang, Zongbin Li, Yuqi Liu, Yuxiao Zhang, Tong Yin, Yang Li

**Affiliations:** 1 Department of Geriatrics, The First Affiliated Hospital of Chongqing Medical University, Chongqing, China; 2 Department of Cardiology, General Hospital of Chinese People’s Liberation Army, No.28 Fu Xing Road, Beijing, 100853 China; 3 Department of Ophthalmology, 958 Hospital of PLA ARMY, Chongqing, China; 4 Department of Cardiology, Beijing Friendship Hospital, Capital Medical University, Beijing, China

**Keywords:** Hypertension, Mitochondria, NADH dehydrogenase subunit 2, Variation, Interaction

## Abstract

**Background:**

The mitochondrial genotype 5178 cytosine/adenine (5178 C > A) within the NADH dehydrogenase subunit-2 gene (ND2) was proved to associate with longevity and predispose resistance to adult-onset diseases. This study aimed to confirm the interactive effects between ND 25178 C > A and clinical risk factors on the susceptibility of essential hypertension in Chinese general population.

**Materials and Methods:**

The relationship between the ND2 5178 C > A variation and the risk of hypertension was investigated in 817 hypertensives and 821 matched normotensives. The interactive effects between ND2 5178 C > A and clinical risk factors were evaluated.

**Results:**

The ND2 5178 A allele was more frequent in normotensives than in hypertensives (32.64% vs. 24.24%; adjusted OR: 0.62, 95% CI: 0.49–0.79, *P* = 1.3 × 10^− 4^). After stratification, the significant association between ND2 5178 C > A and hypertension was found only in current smokers (OR: 0.44, 95% CI: 0.31–0.62), but not in non-current smokers (*p* <  0.01 for interaction). Smoking status (OR: 1.51, 95% CI: 1.11–2.06) and high triglycerides (OR: 1.57, 95% CI: 1.10–2.24) were found independently associated with hypertension only in carriers of 5178 C allele but not in carriers of 5178 A allele.

**Conclusions:**

In conclusion, ND2 5178 A allele could confer a lower risk for essential hypertension in Chinese by the interaction with smoking status. The higher risk of hypertension imposed by smoking and high TG may be altered by ND2 5178 A allele.

**Electronic supplementary material:**

The online version of this article (10.1186/s12881-019-0838-3) contains supplementary material, which is available to authorized users.

## Background

Essential hypertension remains an enormous public health concern, imposing a major burden of morbidity and mortality worldwide [1]. It is reported that 58.3% of deaths from hemorrhagic strokes and 54.5% of deaths from ischemic heart disease could be attributed to hypertension [2]. Hypertension is generally recognized as a multifactorial trait involving interactions among genetic, environmental and demographic factors [3–5]. Although the nuclear genome has been studied extensively for the contribution to hypertension [6], common variations could only explain less than 3% of the variance of blood pressure [7]. Recently, mitochondrial tRNA variations were reported being associated with maternally inherited hypertension in pedigree hypertensive patients, by damaging mitochondrial respiratory function and subsequently leading to the accumulation of reactive oxygen species (ROS), which might involve in the pathogenesis of hypertension [8–14]. However, the contribution of coding genetic variations in the mitochondrial genome has seldom been investigated for their association with hypertension.

Mitochondrial DNA 5178 cytosine/adenine (5178 C > A) (ID number: rs28357984) located in the coding gene of NADH dehydrogenase subunit-2 gene (ND2) has been reported being associated with longevity in Japanese populations. The finding indicated that ND2 5178 C > A predisposed resistance to adult-onset diseases [15]. Experiments in animal models then supported that carrier of ND2 5178 C > A had a lower risk for type I diabetes mellitus, probably owing to the decreased mitochondrial ROS production [16]. Several studies reported the protective effects of ND2 5178 C > A against adult-onset diseases in general populations [17–26], however, most of the studies were performed in Japanese without the validation in ethnic diverse populations. Therefore, it remains obscure for the protective effects of ND2 5178 C > A genotype against adult-onset diseases in human being. As one of the adult-onset diseases, hypertension has been demonstrated in our previous studies to relate closely to mitochondrial variations [8, 11–14]. Therefore, in the present case-control study, we aimed to confirm the association between ND2 5178 C > A and hypertension in Chinese general population. In addition, the influence of clinical risk factors on association between ND2 5178 C > A and susceptibility of hypertension was also evaluated.

## Materials and methods

### Subjects’ recruitment and clinical characteristics

Hypertensives and normotensives were enrolled from Institute of Geriatric Cardiology and Health Examination Center in General Hospital of Chinese People’s Liberation Army from February 2013 to January 2014. All subjects were Chinese-Han, without the inclusion of their first- or second- degree relatives. The written informed consent for clinical evaluations and genetic analysis were obtained from each participant. Inclusion criteria for hypertensives are as follows: an age of hypertension onset between 30 and 59 years; systolic blood pressure (SBP) ≥ 160 mmHg, diastolic blood pressure (DBP) ≥ 95 mmHg or long term antihypertensive treatments; no causes of secondary hypertension (such as chronic renal disease, renal arterial stenosis, primary aldosteronism, coarctation of the aorta, thyroid disorders, Cushing’s syndrome and pheochromocytoma); family (parents or siblings) history of hypertension; cholesterol (TC) < 6.47 mmol/l; triglycerides (TG) < 2.26 mmol/l [27]. Inclusion criteria for normotensives include as follows: SBP ≤ 130 mmHg and DBP ≤ 85 mmHg; no antihypertensive treatments; no family history of hypertension; age ≥ 50 years; TC < 6.47 mmol/l; TG < 2.26 mmol/l. Those who had been diagnosed as coronary heart disease, diabetes, cardiomyopathy, rheumatic heart diseases, valve diseases, congenital heart diseases, stroke, liver dysfunction, renal failure, cancer and pregnancy were excluded from this study. Demographic and clinical data including age, gender, body mass index (BMI), smoking, drinking, TC, TG, low density lipoprotein cholesterol (LDL-C), high density lipoprotein cholesterol (HDL-C), serum creatinine (SCr), blood urea nitrogen (BUN) and fasting plasma glucose (FPG) were recorded for each subject. To analyze the interactive effect between ND2 5178 C > A and the clinical risk factors, subgroups were divided according to the laboratory variables with the thresholds defined by Chinese guidelines for the management of hypertension [28] and life styles including smoking and drinking status. Current smokers were defined according to the National Health Interview Survey and National Survey on Drug Use and Health research [29]. Participants who drank at least twice per month and had lasted for at least 6 months were defined as current drinkers [30]. The protocol of the study was approved by the medical ethics committee of the Chinese People’s Liberation Army General Hospital.

### Genotyping of mitochondrial ND2 5178 C > A

DNA was extracted from 3 ml whole blood of each subject using the QIA amp DNA Mini-Kit (Qiagen, Hilden, Germany). Genotyping of ND2 5178 C > A was performed using the SNaPshot™ kit following the manufacturer’s instruction (Applied Biosystem) and a 9700 Thermalcycler (Applied Biosystem). Primers for the amplification of the target sequence were designed as 5′-TCCTAACTACTACCGCATTCCT-3′ for forward primer; and 5′-GTGGATGGAATTAAGGGTGTT-3′ for reverse primer. The specific procedure for the amplification was detailed elsewhere [31].

### Statistical analysis

Continuous variables were expressed as mean ± SD, and discrete variables expressed as frequency. Clinical characteristics with continuous variables was assessed by the unpaired, 2-tailed Student’s *t*-test, and those with discrete variables were analyzed by Pearson’s χ^2^-test. Association between ND2 5178 C > A and hypertension was assessed by logistic regression analysis. Hypertensive and normotensive status were numerically coded as 1 and 0 respectively. In addition to ND2 5178 genotype, age, gender, BMI, smoking, drinking, TC, TG, LDL-C, HDL-C, SCr, BUN and FPG were all entered in a forward stepwise logistic regression procedure to conduct adjustment. *P*-value < 0.05 was considered statistically significant. Effects of clinical factors on the risk of hypertension in subgroups divided by the genotype were also assessed by logistic regression analysis with forward stepwise method. Variables with P-value < 0.05 were finally considered having significant association with hypertension. The above statistical analysis was performed using the SPSS software package (version 13.0). The interactive effects of the clinical factors on the association between ND2 5178 A allele and hypertension were assessed by the Review Manager (version 5.1) as described below. The odds ratios for hypertension associated with ND2 5178 A allele were analyzed in subgroups. In subgroups divided by each clinical factor, *P* value for heterogeneity was calculated to evaluate the discrepancy between the two subgroups. Chi-squared test was applied to evaluate the heterogeneity, and P for heterogeneity < 0.05 was considered as heterogeneity. In this study, heterogeneity means that the correlation between 5178 C > A and hypertension could be influenced by the clinical factor.

## Results

### Clinical characteristics of participants

A total of 817 hypertensives and 821 normotensives were recruited (Additional file 1). For the baseline clinical characteristics, significant difference could be found for the distribution of BMI, SBP, DBP, TG, HDL-C, SCr, BUN and FPG between hypertensives and normotensives (Table 1).

**Table 1:** Clinical characteristic of hypertensives and normotensives

	Hypertensive (*n* = 817)	Normotensive (*n* = 821)	*P* value
Male, n (%)	616 (75.40)	585 (71.25)	0.06
Age, years	55.08 ± 9.41	55.42 ± 5.32	0.36
BMI, kg/m^2^	26.12 ± 3.39	23.90 ± 2.85	< 0.01
Current smokers, n (%)	291 (35.62)	269 (32.76)	0.22
Current drinkers, n (%)	453 (55.45)	458 (55.79)	0.89
SBP, mmHg	131.72 ± 15.81	112.16 ± 10.02	< 0.01
DBP, mmHg	85.52 ± 11.89	74.54 ± 7.39	< 0.01
TC, mmol/l	4.61 ± 0.85	4.59 ± 0.63	0.58
TG, mmol/l	1.35 ± 0.44	1.18 ± 0.43	< 0.01
LDL-C, mmol/l	2.95 ± 0.80	2.96 ± 0.56	0.91
HDL-C, mmol/l	1.25 ± 0.32	1.35 ± 0.33	< 0.01
SCr, μmol/l	75.00 ± 20.50	72.26 ± 13.15	< 0.01
BUN, mmol/l	5.43 ± 2.13	5.02 ± 1.14	< 0.01
FPG, mmol/l	5.79 ± 1.10	5.41 ± 0.49	< 0.01

*BMI* body mass index, *SBP* systolic blood pressure, *DBP* diastolic blood pressure, *TC* total cholesterol, *TG* triglyceride, *LDL-C* low density lipoprotein cholesterol, *HDL-C* high density lipoprotein cholesterol, *SCr* serum creatinine, *BUN* blood urea nitrogen, *FPG* fasting plasma glucose

### The association between ND2 5178 C > A and hypertension

The frequency of ND2 5178 A allele was significantly lower in hypertensives than in normotensives (24.24% vs. 32.64%, *P* = 1.70 × 10^− 4^). After the logistic regression analysis with the adjustment of the related baseline characteristics, a lower risk for hypertension could be observed in ND2 5178 A allele carriers (adjusted OR: 0.62, 95% CI: 0.49–0.79, *P* = 1.30 × 10^− 4^) (Table 2).

**Table 2:** Relationship between mitochondrial ND2 5178 C > A and hypertension

Genotype	Genotype frequency		OR (95% CI)	*P* value	Adjusted OR^a^ (95% CI)	*P*^a^ value
	Hypertensiven (%)	Normotensiven (%)				
5178 C	619 (75.76)	553 (67.36)	–		–	
5178 A	198 (24.24)	268 (32.64)	0.66 (0.53–0.82)	1.70 × 10^−4^	0.62 (0.49–0.79)	1.30 × 10^− 4^

*OR* odds ratio, *CI* confidence interval. ^a^ Adjusted for age, gender, BMI, smoking, drinking, TC, TG, LDL-C, HDL-C, SCr, BUN and FPG levels

### Interactive effects between ND2 5178 C > A and clinical factors on hypertension

**Fig. 1:**
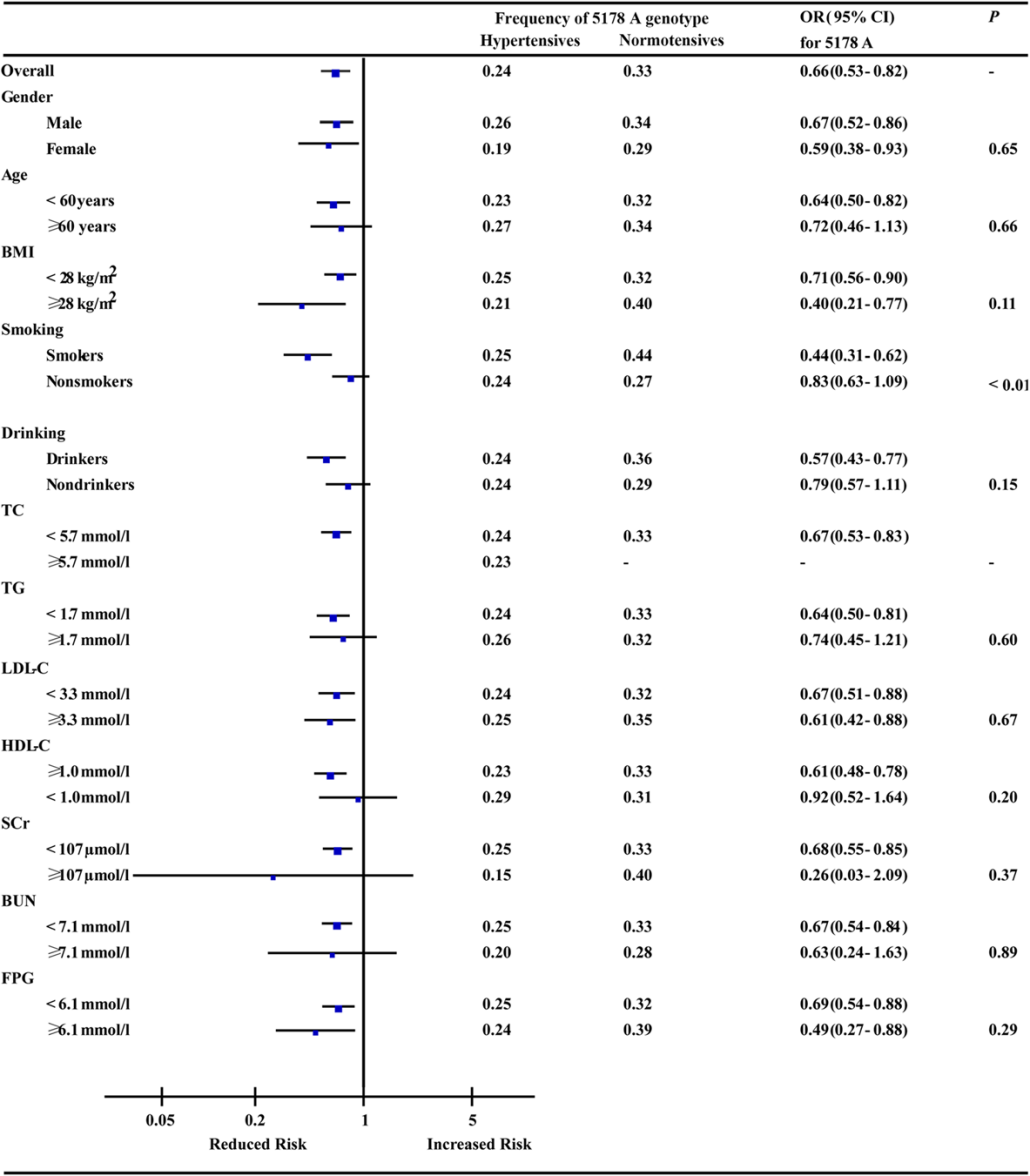
Odds ratios for hypertension associated with 5178 A among subgroups (black squares indicate odds ratios, and horizontal lines indicate 95% CIs)

The interactive effects of the stratified baseline clinical characteristics on the association between ND2 5178 C > A and hypertension were showed in Fig. 1. The association between ND2 5178 A allele and lower risk of hypertension was not significantly modified by the interactive effects exerted by gender, age, BMI, drinking, TC, TG, LDL-C, HDL-C, SCr, BUN and FPG levels (*P*_*for heterogeneity*_ > 0.05). However, the lower risk for hypertension in ND2 5178 A allele carriers was found only in current smokers (OR: 0.44, 95% CI: 0.31–0.62), but not in non-current smokers (OR: 0.83, 95% CI: 0.63–1.09, *P*_*for heterogeneity*_ <  0.01) (Fig. 1). The interactive effects of ND2 5178 C > A on the traditional risk factors of hypertension in the present population were shown in Table 3. Current smoking status conferred a higher risk for hypertension only in ND2 5178 C allele carriers (OR: 1.51, 95% CI: 1.11–2.06, *P* = 0.01), but not in ND2 5178 A allele carriers (OR: 0.64, 95% CI: 0.39–1.04, *P* = 0.07). In addition, TG was an independent risk factors for hypertension only in subjects with ND2 5178 C (OR: 1.57, 95% CI: 1.10–2.24, *P* = 0.01), but not in those with ND2 5178 A allele (OR: 0.73, 95% CI: 0.38–1.41, *P* = 0.35).

**Table 3:** Effects of clinical factors on the risk of hypertension according to mitochondrial ND2. 5178 C > A genotype

	ND2. 5178 C carriers (*n* = 1172)		ND2. 5178 A carriers (*n* = 466)	
	OR^a^ (95% CI)	*P*^a^ value	OR^a^ (95% CI)	*P*^a^ value
Male, n (%)	0.83 (0.54–1.25)	0.37	1.31 (0.63–2.71)	0.47
Age, years	0.99 (0.97–1.01)	0.33	1.00 (0.97–1.03)	0.94
BMI, kg/m^2^	1.24 (1.18–1.30)	< 0.01	1.19 (1.10–1.29)	< 0.01
Current smokers, n (%)	1.51 (1.11–2.06)	0.01	0.64 (0.39–1.04)	0.07
Current drinkers, n (%)	0.92 (0.67–1.27)	0.63	0.63 (0.38–1.07)	0.09
TC, mmol/l	2.02 (1.26–3.25)	< 0.01	10.59 (3.05–36.79)	< 0.01
TG, mmol/l	1.57 (1.10–2.24)	0.01	0.73 (0.38–1.41)	0.35

*OR* odds ratio, *CI* confidence interval, *BMI* body mass index, *TC* total cholesterol, *TG* triglyceride

^a^Adjusted for age, gender, BMI, smoking, drinking, TC, TG, LDL-C, HDL-C, SCr, BUN and FPG levels

## Discussion

The present study showed that ND2 5178 C > A was associated with a significantly lower risk for hypertension in the present Chinese general population. However, this protective effect of ND2.5178 A allele was observed particularly in current smokers but not in nonsmokers. On the other hand, current smoking status and elevated TG were independent risk factors for hypertension only in 5178 C allele carriers but not in ND2 5178 A allele carriers. It indicated that ND2 5178 A allele could confer a lower risk for essential hypertension by the interaction with smoking status. The higher risk of hypertension imposed by smoking and high TG may be altered by the variation. To the best of our knowledge, the present study confirmed for the first time that the ND2 5178 A allele could protect against hypertension in Chinese general population by the interaction with clinical risk factors.

Our present study identified a significantly lower risk of hypertension in ND2 5178 A allele carriers based on a total of 817 hypertensives and 821 normotensives. The paradox association between ND2 5178 C > A and hypertension was reported previously [19, 20, 32]. One study in Japanese men (*n* = 398) showed the frequency of hypertension was higher in ND2 5178 C allele carriers than in 5178 A allele carriers [19]. Whereas, another study in Japanese women (*n* = 412) found higher diastolic blood pressure in carriers of ND2 5178 A allele than in carriers of ND2 5178 C allele [20]. There was no statistical difference between sexes in this present study. The difference on race and sample size might partly explain the disparity. However, much more evidences would be needed in future research. More importantly, clinical risk factors rather than gender might have more impact on such association between ND2 5178 C > A and hypertension in Chinese population.

Thus, we further analyze the association between ND2 5178 C > A and hypertension based on stratification according to clinical risk factors. The protective effect of ND2 5178 A allele on hypertension was found only in current smokers but not in non-current smokers. As we know, smoking-induced generation of ROS has a strong association with hypertension. Mice exposed to smoking showed increased ROS and consequently elevated BP [33]. The 5178 C > A variation results in the amino acid change of leucine to methionine in ND2 gene. Methionine residues have been proved to be the main oxidation site within proteins [34]. Thus, we speculate that the protective effect presented by methionine residues resulted from ND2 5178 C > A variation might be particularly obvious in current smokers. However, we did not observe the interaction between ND2 5178 C > A and drinking status on the risk for hypertension as previously reported [19]. The difference might attribute to the diverse definition for drinking and the gender difference of participants. The interaction between ND2 5178 C > A and drinking status on risk for hypertension was detected previously only in male subjects. Whereas, both male and female subjects were recruited in our study. Considering the less drinking habit in women, it would be more difficult to observe such interaction in the present study.

The association between either smoking or TG and hypertension was apparent only in subjects carrying ND2 5178 C allele, but not in those carrying ND2 5178 A allele. It implicated that the risk of hypertension induced by smoking and TG might be overcome by the protective effect of ND2 5178 A allele. Another possible explanation may relate to the lower concentration of TG in ND2 5178 A allele carriers [35]. Therefore, the association between TG and hypertension in subjects with ND2 5178 A allele may not be obviously observed.

There are still some limitations in the present study. The main limitation is that the subjects in the present study were recruited from the single center, and the sample size was not large enough, particularly considering the fairly high number of subgroup analyses that were conducted. The statistical power was weakened by this limitation to some extent. The conclusion of this study would be more convincing if some subgroup observations, such as the conditional association between the protective ND2 5178 A allele and hypertension only within current smokers, could be validated in an independent cohort. In addition, the definite mechanisms for the protection of hypertension in ND2 5178 A allele carriers still remains to be illuminated in further investigation.

## Conclusion

In conclusion, mitochondrial ND2 5178 C > A variation contributed to a lower risk of hypertension in Chinese. The association between ND2 5178 C > A variation and lower risk for hypertension in Chinese was influenced by smoking status. The higher risk of hypertension imposed by smoking and high TG may be altered by the variation.

## Additional file

Additional file 1: Raw data. (XLS 426 kb)

## Data Availability

The datasets used and analyzed during the current study are available from the corresponding author upon reasonable request.

## Abbreviations

BMI: triglycerides
BUN: blood urea nitrogen
DBP: diastolic blood pressure
FPG: fasting plasma
HDL-C: high density lipoprotein cholesterol
LDL-C: low density lipoprotein cholesterol
ND2: NADH dehydrogenase subunit-2 gene
ROS: reactive oxygen species
SBP: systolic blood pressure
SCr: serum creatinine
TC: cholesterol
TG: triglycerides
